# Clinical and radiographic evaluation of maxillary central incisors exposure in patients undergoing maxillary advancement

**DOI:** 10.1590/2177-6709.20.6.052-059.oar

**Published:** 2015

**Authors:** Guilherme dos Santos Trento, Felipe Bueno Rosettti Bernabé, Delson João da Costa, Nelson Luis Barbosa Rebellato, Leandro Eduardo Klüppel, Rafaela Scariot

**Affiliations:** 1 Resident in Oral and Maxillofacial Surgery, Universidade Federal do Paraná (UFPR), Curitiba, Paraná, Brazil; 2 Specialist in Oral and Maxillofacial Surgery, Universidade Federal do Paraná (UFPR), Curitiba, Paraná, Brazil; 3 Professor, Universidade Federal do Paraná (UFPR), Residency program in Oral and Maxillofacial Surgery, Curitiba, Paraná, Brazil; 4 Professor, Universidade Positivo, Undergraduate program in Dentistry, Curitiba, Paraná, Brazil.

**Keywords:** Orthognathic surgery, Maxilla, Incisor, Esthetics

## Abstract

**Introduction:**

Patients with dentofacial deformities may undergo orthodontic or orthodontic-surgical treatment. Both modalities can affect esthetics.

**Objective:**

This study aims to evaluate clinical and radiographic changes in exposure of maxillary central incisors occurring after orthognathic surgery for maxillary advancement.

**Methods:**

A total of 17 patients who underwent orthognathic surgery for maxillary advancement between September, 2010 and July, 2011 were selected. Exposure of maxillary central incisors was evaluated clinically and by lateral cephalograms. Measurements were taken one week before and three months after surgery. Data were paired in terms of sex, age, nasolabial angle, height and thickness of the upper lip, the amount of maxillary advancement, clinical exposure and inclination of maxillary central incisor by statistical tests (CI 95%).

**Results:**

After maxillary advancement, incisor clinical exposure had increased even with relaxed lips and under forced smile. Moreover, there was a mean increase of 23.33% revealed by lateral cephalograms. There was an inverse correlation between upper lip thickness and incisors postsurgical exposure revealed by radiographic images (*p* = 0.002).

**Conclusions:**

Significant changes in the exposure of maxillary central incisors occur after maxillary advancement, under the influence of some factors, especially lip thickness.

## Resumo

**Introdução:**

pacientes portadores de deformidades dentofaciais podem submeter-se a tratamento ortodôntico ou ortodôntico-cirúrgico. Ambos podem modificar a estética do paciente.

**Objetivo:**

esse estudo tem por objetivo avaliar, clinicamente e radiograficamente, as mudanças na exposição dos incisivos centrais superiores em pacientes submetidos à cirurgia ortognática de avanço de maxila.

**Métodos:**

foram selecionados 17 pacientes submetidos à cirurgia ortognática de avanço maxilar no período de setembro de 2010 a julho de 2011. A exposição dos incisivos centrais superiores foi avaliada clinicamente e por meio de radiografias cefalométricas em norma lateral. Essas medidas foram tomadas uma semana antes e três meses depois da cirurgia. Os dados foram, por meio de testes estatísticos (CI 95%), correlacionados por sexo, idade, ângulo nasolabial, altura e espessura do lábio superior, quantidade de avanço maxilar, exposição clínica e inclinação dos incisivos centrais superiores.

**Resultados:**

após o avanço maxilar, houve um aumento da exposição clínica dos incisivos tanto com o lábio superior relaxado quanto sob sorriso forçado. Além disso, obteve-se um aumento médio de 23,33% na exposição dos incisivos nas radiografias cefalométricas em norma lateral. Houve correlação inversa entre a espessura do lábio superior e a exposição pós-cirúrgica dos incisivos nas imagens radiográficas (p = 0,002).

**Conclusão:**

mudanças significativas na exposição dos incisivos centrais superiores ocorrem após o avanço maxilar, sob influências de certos fatores, especialmente a espessura do lábio superior.

## INTRODUCTION

Severe malocclusion requires combined treatment of surgery and Orthodontics. Less severe dentofacial deformities can be treated only by orthodontic treatment.^1^ Changes in the facial skeleton produced by this treatment modality affect not only the bones of the facial skeleton, but also the relationship between hard and soft tissues of the face.^2^ The most widely used technique for repositioning the maxilla is Le Fort I osteotomy which can be used for correction of vertical, anteroposterior and transverse problems that involve the maxilla by means of osteotomies across the anterior and lateral walls of this structure.^3^ In cases of Class III malocclusion, maxillary advancement aims to correct the bite, improve facial esthetics and harmonize the facial profile. Therefore, it is important for the clinician to be able to predict soft tissue changes resulting from alterations of hard tissues.^4^ Soft tissue changes resulting from maxillary advancement via Le Fort I osteotomy have been reported to be between 33% and 100%.^5^^,^ 6 Studies that describe the influence of soft tissue surgical corrections are limited.^7^^-^ 10 Nevertheless, some studies have shown that changes in the soft tissues of the lips are influenced by the magnitude and direction of the jaw segment during surgery,^11^ and mainly by tone and lip thickness.^5^^,^ 12 ^,^ 13 ^,^ 14 In the case of impaction, and with posterior or anterior movement of the maxilla, it was found that the nasolabial angle is increased despite a wide variation in tissue responses.^9^^,^ 15 Bundgaar, Melsen, and Terp^7^ hypothesized that angular changes may be related to muscle function on the site of osteotomy, and assessment of patient's muscle pattern could be important for predictive tracing of hard and soft tissues. Stella et al,^16^ in order to assess the predictability of changes in the soft tissue of the upper lip as a result of maxillary advancement by the Le Fort I technique, selected 20 adult patients with a follow-up of six months. Patients were subdivided into two groups based on lip thickness: Group 1 (lips between 10 and 17-mm thick) and Group 2 (greater than 17-mm thick). Most patients showed a reduction in thickness of the upper lip, and presented no increase in thickness. The reduction of lip thickness was greater than 25% in most patients. It was further stated that clinically relevant correlations cannot be made between the change of soft tissues and bone advancement; however, when the reference is the thickness of the upper lip, there is a better relationship between these two variables.^15^^,^ 16 In a retrospective cephalometric study, Van Butsele et al^17^ evaluated soft and hard tissue ratios in relation to maxillary advancement. The authors concluded that, for each millimeter of maxillary advancement, the upper lip moved upward in almost 30% the amount of advancement, in addition to having an elongation of 1.7 mm. Del Santo et al^18^ evaluated 19 patients undergoing Le Fort I osteotomy in order to study changes in the lips. The authors concluded that significant horizontal changes occur in the upper lip when the maxilla is moved significantly anteroposteriorly at a ratio of 0.6 : 1. This is because the vertical changes of the upper lip only occur when there is a significant change in the anteroposterior position of the maxillary basal bone. Given the above statement, the objective of this study was to evaluate the clinical and radiographic changes, with exposure of maxillary central incisors, occurring after maxillary advancement.

## MATERIAL AND METHODS

Sample selection: A total of 17 patients were selected to undergo orthognathic surgery for maxillary advancement in the Department of Oral and Maxillofacial Surgery, Universidade Federal do Paraná, in the period of September, 2010 to July, 2011. All patients (aged 18 or older) included in the study had Class III malocclusion and underwent maxillary advancement alone or combined with mandibular surgery, with previous orthodontic decompensation. Those who did not get V-Y closure on the upper lip, did not present central incisors and did not attend postoperative control were excluded from the sample. This research was approved by the Ethical Research Committee on Human Beings at the Human Health Department under number CEP/ D: 921.046.10.05 and CAAE: 0033.0.091.000-10. All patients signed an informed consent form.

Clinical analysis: All clinical measurements were performed with patients seated and with their head in natural position. Clinical analysis of maxillary central incisors exposure with a relaxed lip and under forced smile was performed one week before surgery and three months after surgery. Clinical measurements were taken with the aid of a digital caliper (Vonder^TM)^. These measurements consisted of the distance between the lowest upper lip point and the incisal edge of maxillary incisors.

**Figure 1:**
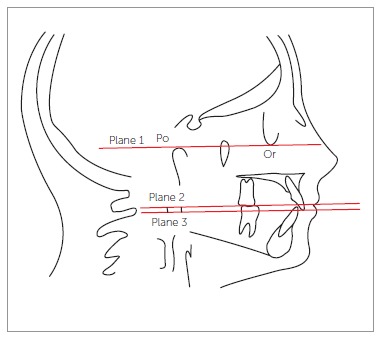
- Radiographic analysis of exposure of maxillary central incisor. Plane 1: Frankfort horizontal plane (FH). Plane 2: Plane passing through stomion superius parallel to FH. Plane 3: Plane passing through the edge of maxillary central incisor parallel to FH. Po = Porion. Or = Orbitale.

**Figure 2:**
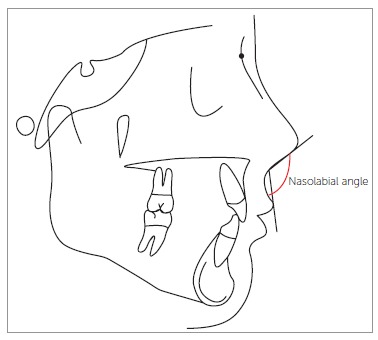
- Measure of nasolabial angle (angle formed by a line tangent to the columella through the subnasale and by a line tangent to the upper lip passing through the labial superius).

**Figure 3:**
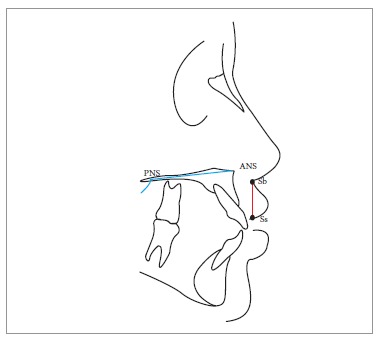
- Measure of upper lip height (plane passing through subnasale and stomion superius). PNS = Posterior nasal spine. ANS = Anterior nasal spine. Sb = Subnasale. Ss = Stomion superiorius.

**Figure 4:**
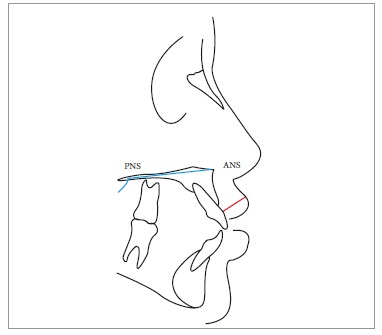
- Measure of upper lip thickness. PNS = Posterior nasal spine. ANS = Anterior nasal spine.

**Figure 5:**
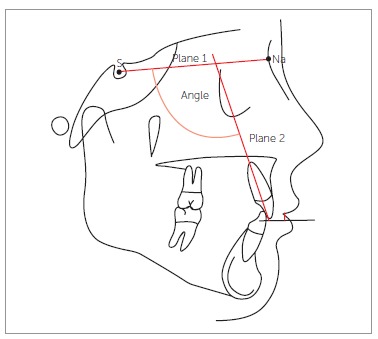
- Inclination of the upper central incisor to the Sella-Nasion plane.

**Figure 6:**
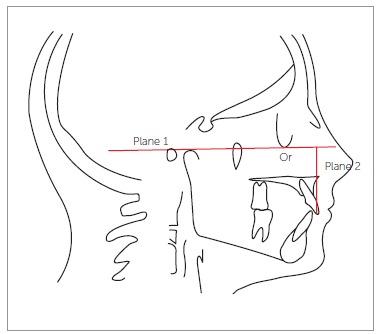
- Measure from the incisal edge to the Frankfort horizontal plane. Plane 1: Frankfort horizontal plane (FH). Plane 2: Plane perpendicular to FH through the incisal edge of the upper central incisor. Po = Porion. Or = Orbitale.

Radiographic analysis: Lateral cephalograms were performed one week before and three months after the surgical procedure. All radiographs were performed by the equipment Orthophos model 90 KV/12 mA (Siemens^TM^, Germany) located at the Department of Dental Radiology. All radiographs were taken with the lips at rest and in natural head position. Presurgical and postsurgical cephalograms were traced and analyzed at three different time intervals in order to perform intra-examiner calibration, and through the intraclass correlation coefficient (pre ICC = 0.984 and post ICC = 0.993), which allowed radiographic interpretation to be conducted by the same examiner. After identifying the cephalometric landmarks of interest to the study, we assessed exposure of maxillary central incisors in pre and postsurgical cephalograms. Three planes were traced from the cephalometric landmarks: Frankfort horizontal plane passing through the porion (higher point in the contour of the ear canal) and orbitale (lowest point of the lower edge of the contour of the orbital cavity); a plane passing through the incisal edge of maxillary incisor; and a plane passing through the stomion superius. These last two planes were traced parallel to Frankfort horizontal plane. Thus, after the three planes had been outlined, a measurement was made using Rickets' rule from the incisal edge of the maxillary central incisor to the stomion superius (distance between the plane passing through the incisal edge and the plane passing through the stomion superius) (Fig 1). Moreover, the nasolabial angle in the presurgical radiographs was traced and measured (angle formed by a line tangent to the columella through the subnasale landmark and by a line tangent to the upper lip passing through the labial superius) (Fig 2). The height (line joining subnasale and stomion superius) and the width of the upper lip in the presurgical radiographs were also traced and measured (Figs 3 and 4). Finally, inclination of maxillary central incisors before and after surgery were measured by the angle formed by the long axis of the maxillary central incisor to the sella-nasion line (plane passing through nasion and sella landmarks) (Fig 5). Because surgeries were performed by different surgeons, measurements were also taken on pre- and postoperative radiographs to ensure that there were no vertical movements of the maxillary segment. Thus, the Frankfort horizontal plane and a line perpendicular to this plane through the incisal edge were traced. Measurement was performed by the distance from the incisal edge to the Frankfort horizontal plane (Fig 6).

Plane 1: Plane passing through sella and nasion. Plane 2: Plane passing through the long axis of the upper central incisor. S = Sella. Na = Nasion.

Statistical analysis: Results were submitted to descriptive and statistical analysis. Statistical evaluation was performed by frequency analysis and specific statistical tests using the Statistical Package for Social Sciences^TM^ (version 15.0; SPSS Inc., Chicago, IL, USA) with a 95% confidence interval.

## RESULTS

The sample consisted of 17 patients (14 females and 3 males). Sex was not correlated with increased clinical and radiographic exposure of maxillary central incisors after maxillary advancement (*p* = 0.423). Patients had a mean age of 23 years in the sample (18-41). Age was not correlated with increased clinical and radiographic exposure of maxillary central incisors after orthognathic surgery (*p* = 0.650). Table 1shows all values found in the clinical and radiographic exposure of maxillary central incisors both pretreatment and post-treatment. Mean clinical exposure of maxillary central incisors with relaxed lip was 3.20 mm (0 - 7 mm) at the preoperative stage and 4.21 mm (0 - 6.60 mm) at the postoperative stage. Thus, there was a mean increase of 31% after maxillary advancement. Mean clinical exposure of maxillary central incisors under forced smile was 8.30 mm (4.50 - 14.10 mm) at the preoperative stage and 9.16 mm (5.10 - 15.02 mm) at the postoperative stage. There was a statistical association between pre and postoperative measurements (Wilcoxon test / *p* = 0.001 - CI 95%). Mean exposure of maxillary central incisors in lateral cephalograms (presurgical) was 3.00 ± 1.46 mm, while postsurgical mean was 3.70 ± 1.59 mm. Thus, there was a mean increase in the exposure of central incisors of 0.70 mm which corresponds to 23.33%. The variables of exposure were also correlated with these same teeth on preoperative and postoperative radiographs (paired Student's t-test / *p* < 0.001 - CI 95%). The mean amount of maxillary advancement was 5.11 mm. There was no statistical association between increased radiographic exposure of maxillary central incisors and the amount of maxillary advancement (*p* = 0.951). Mean lip thickness was 14.05 ± 2.58 mm. There was a statistically significant correlation between increased exposure of maxillary central incisors and lip thickness after maxillary advancement in lateral cephalograms (*p* = 0.002 / r = 0.696 - CI 99%). In this study, the nasolabial angle had a mean value of 101.70 ± 13.30°. This angle is not related to increased exposure of maxillary central incisors in any measures revealed by radiographic images (*p* = 0.398 - Spearman Correlation Coefficient - CI 95%). Mean lip height was 20.00 ± 2.29 mm. There was no statistically significant correlation between lip height and increased radiographic exposure of these teeth after surgery (Pearson's Correlation Coefficient - *p* = 0.357). Mean maxillary incisors inclination was 113.35 ± 8.39° preoperatively. This variable was not correlated with increased radiographic exposure of incisors (*p* = 0.533). Postoperative inclination of maxillary incisors ranged as from an average of 114.88 ± 7.50°. There was no association between postoperative inclination and radiographic exposure of incisors after surgery (*p* = 0.814). It was not possible to associate inclination of maxillary central incisors preoperatively with postoperatively by means of paired Student's t-test (*p* = 0.059), which had an average increase of one degree between these two surgical times. There was no statistical association between increased radiographic exposure of maxillary central incisors and the difference in inclination before and after surgery (*p* = 0.259). All results can be seen in Table 1.

**Table 1:** - Values of clinical and radiographic exposure of maxillary central incisors, pre and postoperatively; maxillary advancement; upper lip thickness preoperatively; nasolabial angle preoperatively; preoperatively height of the upper lip, and inclination of maxillary central incisor in pre and postoperative periods.

		**Mean ± SD**	**Median (Min-Max)**	**p (CI = 95%)**
Radiographic exposure of maxillary central incisor (mm)	Preop.	3.00 ± 1.46	---	< 0.001*
	Postop.	3.70 ± 1.59		
Relaxed lip (mm)	Preop.	---	3.20 (0 - 7.00)	= 0.004**
	Postop.		4.21 (0 - 6.61)	
Forced smile (mm)	Preop.	---	8.30 (4.50 - 14.10)	= 0.002**
	Postop.		9.16 (5.10 - 15.02)	
Maxillary advancement (mm)	---	---	5.00 (4.00 - 8.00)	---
Lip thickness (mm)	Preop.	14.05 ± 2.58	---	---
Nasolabial angle (degrees)	Preop.	102.29 ± 12.73	---	---
Lip height (mm)	Preop.	20.00 ± 5.36	---	---
Maxillary central incisor inclination (degrees)	Preop.	113.35 ± 8.39	---	= 0.059*
	Postop.	114.88 ± 7.50	---	= 0.059*

*Paired Student's t-test; **Wilcoxon Test.

## DISCUSSION

It is essential to be able to predict postoperative hard tissue and facial profile changes resulting from orthognathic surgery in order to achieve functional and esthetic success of the procedure.^13^^,^ 19 ^,^ 20 ^,^ 21 The literature suggests that the etiology of soft tissue changes is postsurgical edema, increased support of bone tissue and the elevation of periosteum and muscles near the nose without correct repositioning.^13^ Facial changes in patients undergoing orthognathic surgery performed in the upper jaw are multifactorial.^15^^,^ 19 If there was a pattern to predict the amount of exposure of maxillary central incisors after orthodontic-surgical combined treatment, there would be an ideal preoperative predictability. However, it is difficult to have a standard or a universal method to measure the exposure of teeth with relaxed lips and at smiling because of the number of variables that may be associated with it, such as the degree of muscle activity, individual diversity factors and age.^22^ In addition, there are differences in the studies regarding the selection of the sample, namely: the inclusion of patients with birth defects or syndromes in the same sample of patients with facial deformity; the use of different radiological equipment to perform presurgical and postsurgical radiographs; the difficulty maintaining the correct position of the head and patients' lips at the time of radiograph; the exclusion or non-exclusion of segmental surgeries in the sample; only one or multiple motion vectors in the maxilla in this sample; using the same technique of incision and suture in the sample; in addition to osteoplasty (e.g., recontour of the anterior nasal spine) and follow-up time.^13^^,^ 18 ^,^ 19 Other prominent factors are the complexity of anatomical structures in this region of the face, the technical difficulty of correctly visualizing the outline on the radiograph, the absence of a specific and unique methodology as the means of performance, and comparison of tracings. Due to this diversity of factors that can alter the results, there is a limitation in comparing studies.^18^ Although women tend to display greater maxillary incisor exposure at rest and movement than men,^23^ the study found no relationship of sex with increased exposure of maxillary central incisors after maxillary advancement. It is worth noting that the research sample had a small number of men (n = 3), which interfered in the analysis of results. Lee, Bailey, and Proffit^8^ claimed that the physiological variation of age and loss of muscle tone may explain the difference between movement of soft and hard tissues. Younger adults with lack of dentoalveolar support do not show facial concavity, which is usually associated with an older age group. Tonicity and thickness of soft tissues are considered to be responsible for this difference.^19^ Flexibility of soft tissues, especially the lips, is directly influenced by tone and thickness.^12^In this study, age was not related to increased exposure of maxillary central incisors after surgery, since the vast majority of patients were young adults (20 to 30 years old). In most patients who underwent maxillary advancement alone or combined with another procedure in the mandible, the results indicated an increase in radiographic exposure of maxillary central incisors after orthognathic surgery (23.33%). There are two factors described in the literature that may influence this condition: soft tissue changes after orthognathic surgery and changes in bone and tooth structure itself. Thus, with regard to a potential change of soft tissues, we consider in this study some factors that may contribute to this increase in exposure of maxillary central incisors after maxillary advancement as far as the nasolabial angle, height, and thickness of the lips. With regard to a potential change of hard tissue, the amount of maxillary advancement, the inclination of maxillary central incisors before and after surgery, and the difference in inclination of these same structures before and after surgery were considered. According to the results of this study, only soft tissue changes influenced the increased exposure of incisors after maxillary advancement. Soft tissue changes after maxillary advancement may include changes in the positioning of the apex of the nose and nasolabial angle.^15^ The literature shows an increase in the nasolabial angle of 1.20° with anterior repositioning of the maxilla and a mean value of 0.65° for every 1 mm of advancement, although there is a wide variation in tissue response, some patients show an increase,^9^^,^ 15 ^,^ 19 while others show a reduction^12^ in the postsurgical period. Bundgaar, Melsen, and Terp^7^ hypothesized that an angular change may be related to muscle function on the site of the osteotomy.^7^ Therefore, this change in the nasolabial angle would influence exposure of maxillary central incisors, which did not occur in this study. Of the variables analyzed in soft tissues, lip thickness was the most important, as there was a statistically significant correlation between this variable and the radiographic measures. At the same time, it is known that the clinical measurement of a muscular component is unattainable in practice. Radiograph measurements depend on the positioning of the lips during the radiographic procedure which introduces the same significant variation.^8^^,^ 10 ^,^ 20 Considering natural lip thickness, the literature shows that lips with thickness greater than 17 mm have a smaller effect in relation to the movement of maxillary advancement; however, the opposite occurs with thinner lips in comparison to those that had excellent correlation.^24^ Thinner lips tend to expose more of incisors after maxillary advancement, which can be explained by the fact that these lips follow maxillary advancement to a greater degree compared to thicker lips.^5^^,^ 13 ^,^ 14 It is also known that a thick lip can absorb the upper jaw amount of advancement by distension,^18^ in addition to having a firmer grip on the base of the nose, which prevents vertical and horizontal movements of the upper lip in response to maxillary movement.^24^ In our study, we did not find any relation between the preoperative height of the upper lip and increased radiographic exposure of maxillary central incisors after maxillary advancement. Although we found, in the literature, that shorter lips tend to have greater vertical movement after surgery,^25^ small changes were observed in the vertical alteration of the lip with insignificant statistical correlations after maxillary advancement.^9^^,^ 21 Additionally, recent cephalometric investigations have found that movement of hard and soft tissues after orthognathic operations were strongly correlated horizontally but not vertically, and the position of the lips could not be predicted accurately.^12^ Regarding the influence of hard tissues that concern the increased exposure of maxillary central incisors after maxillary advancement, we evaluated the amount of maxillary advancement. This anterior movement is accompanied by a vertical and horizontal movement that influences the exposure of incisors.^17^^,^ 20 In our study, the amount of maxillary advancement was not statistically significant, since the vast majority of advances were of the same size, between 4 mm (47%) and 5 mm (23%). Another factor that could influence the final exposure of maxillary incisors could be an increased inclination of the incisor after orthognathic surgery for maxillary advancement, since orthodontic movement of anterior teeth can influence and result in a change of upper lip position, thus influencing the exposure of maxillary central incisors.^19^ It is worth noting that the option to measure the inclination of maxillary central incisors, before and after surgery, was based on the fact that the second measurement was performed three months after the procedure, which could lead to a biased result if there was a change in the inclination of incisors by orthodontic movement, thus influencing the exposure of maxillary incisors after surgery. In our study, the difference between inclination (pre- and postsurgical period) of these teeth was not significant. There is no relationship between the inclination of maxillary incisors by preoperatively increasing their exposure after surgery. There was also no relationship between the difference in inclination before and after surgery of maxillary incisors and increased exposure of these teeth after surgery.

## CONCLUSIONS

1) Significant clinical change in the exposure of maxillary central incisors occurs after maxillary advancement, with a mean increase of 31% with a relaxed upper lip and 10.36% under forced smile.
2) Significant radiographic change in the exposure of maxillary central incisors occurs after maxillary advancement, with a mean increase of 23.33% in lateral cephalograms.
3) Increased exposure of maxillary central incisors after maxillary advancement is mainly influenced by upper lip thickness. Thin lips tend to expose more of incisors after maxillary advancement, while thicker lips expose less due to their greater adherence to the base of the nose and by presenting more consistency.
4) There is a need for further studies relating to the change in exposure of maxillary central incisors after maxillary advancement.
